# Bacteriophage T4 capsid as a nanocarrier for Peptide-N-Glycosidase F immobilization through self-assembly

**DOI:** 10.1038/s41598-019-41378-9

**Published:** 2019-03-19

**Authors:** Liang Zhang, Pei Wang, Chang Wang, Yike Wu, Xiaojun Feng, He Huang, Lujing Ren, Bi-Feng Liu, Song Gao, Xin Liu

**Affiliations:** 1 Jiangsu Key Laboratory of Marine Bioresources and Environment, Huaihai Institute of Technology, Lianyungang, 222005 China; 2 Britton Chance Center for Biomedical Photonics at Wuhan National Laboratory for Optoelectronics – Hubei Bioinformatics & Molecular Imaging Key Laboratory, Systems Biology Theme, Department of Biomedical Engineering, College of Life Science and Technology, Huazhong University of Science and Technology, Wuhan, 430074 China; 3 Jiangsu Marine Resources Development Research Institute, Lianyungang, 222005 China; 4 Co-Innovation Center of Jiangsu Marine Bio-industry Technology, Huaihai Institute of Technology, Lianyungang, 222005 China; 5 Yugong Biolabs, Inc., Lianyungang, 222000 China; 6 School of Pharmacy, Nanjing Tech University, Nanjing, 211816 China; 7 College of Biotechnology and Pharmaceutical Engineering, Nanjing Tech University, Nanjing, 211816 China

## Abstract

Enzyme immobilization is widely applied in biocatalysis to improve stability and facilitate recovery and reuse of enzymes. However, high cost of supporting materials and laborious immobilization procedures has limited its industrial application and commercialization. In this study, we report a novel self-assembly immobilization system using bacteriophage T4 capsid as a nanocarrier. The system utilizes the binding sites of the small outer capsid protein, Soc, on the T4 capsid. Enzymes as Soc fusions constructed with regular molecular cloning technology expressed at the appropriate time during phage assembly and self-assembled onto the capsids. The proof of principle experiment was carried out by immobilizing *β*-galactosidase, and the system was successfully applied to the immobilization of an important glycomics enzyme, Peptide-N-Glycosidase F. Production of Peptide-N-Glycosidase F and simultaneous immobilization was finished within seven hours. Characterizations of the immobilized Peptide-N-Glycosidase F indicated high retention of activity and well reserved deglycosylation capacity. The immobilized Peptide-N-Glycosidase F was easily recycled by centrifugation and exhibited good stability that sustained five repeated uses. This novel system uses the self-amplified T4 capsid as the nanoparticle-type of supporting material, and operates with a self-assembly procedure, making it a simple and low-cost enzyme immobilization technology with promising application potentials.

## Introduction

Enzymes are capable of accelerating biochemical reactions in a highly efficient and specific manner. Over the past ten years, as a practical and environmental friendly alternative to conventional chemical methods, enzyme-based biocatalysis has contributed to fields and industries including pharmaceutical, biofuel production, chemical, food, cosmetic, textile, paper, environmental monitoring, and disease diagnostics^1–4^. Nevertheless, use of enzymes in industrial applications usually comes along with drawbacks like high cost, poor operational stability, and challenges in recovery and reuse^5–7^. Towards these drawbacks, improvements of enzymes are constantly pursued. Enzyme immobilization provides improved long-term operational stability and simplified enzyme recycling and downstream processing, thus has been broadly applied in biocatalysis^5,7,8^.

Enzyme immobilization techniques include adsorption, covalent binding, affinity immobilization, and entrapment. Selection of an appropriate technique requires the consideration of a number of factors such as overall enzymatic activity, enzyme deactivation and regeneration characteristics, immobilization cost, and desired enzyme property after immobilization^7^. Affinity immobilization utilizes highly specific interactions, gives good control of the orientation of immobilized enzymes, and allows minimal conformational changes. These properties result in high retention of the immobilized enzyme activity^5,7^. Moreover, the affinity immobilization procedure can be used to simultaneously purify the enzyme^9^. Hence affinity immobilization is advantageous over the other techniques in many circumstances.

Selection of the supporting materials is another fundamental factor. In recent years, nanoparticles drew increasing attention because their inherently large surface area enhanced enzyme effectiveness, while immobilizing enzymes onto planar surfaces usually reduced the activity^10–13^. However, their application is hampered by high cost. In fact, high cost of materials, whether nano or normal, is the major reason why commercialization of immobilized enzymes is still at a slower pace. Technology necessary to apply the fixation of enzymes onto the supporting materials also greatly increases the cost^5,7^. Researchers are making arduous efforts to develop simple and stable enzyme immobilization methods that could bring down the cost of immobilized enzymes.

Bacteriophage T4 has served as an excellent model and a primary tool in molecular biology research. The T4 capsid is a 120 nm-long, 86 nm-wide icosahedron that is composed of two essential capsid proteins: a major capsid protein, gp23, and a vertex protein, gp24. Two non-essential proteins, Soc, the small outer capsid protein, and Hoc, the highly antigenic outer capsid protein, decorate the capsid surface at the late stage of capsid assembly^14,15^. There are approximately 870 molecules of the Soc protein (9 kDa) and 155 copies of Hoc protein (39 kDa) assembled on one capsid, providing the phage with survival advantages^16–19^. Under laboratory conditions, however, Soc and Hoc are completely dispensable, making it easy to engineer the T4 capsid surface^16,20^. T4 capsid has been used in a wide range of applications such as peptide library construction and vaccine development^21–25^. Antigens as Soc- or Hoc- fusions were assembled on T4 capsids through affinity binding and exhibited excellent immunization stimulations, indicating stable assembly of exogenous proteins on T4 capsids and good persistence of structures and activities^26–29^. Researchers also attached affinity purification tags (e.g. glutathione S-transferase) onto the T4 capsids through Soc- or Hoc- fusions, and successfully used the tags to purify the phage on standard affinity resins^30,31^. This is another demonstration of stable attaching of proteins or domains onto T4 capsid in the active conformation. The T4 capsid should be a well suited nanoparticle for affinity immobilization of enzymes.

In this study, a novel self-assembly immobilization system has been developed using the T4 capsid as a nanocarrier. The concept of self-assembly on T4 capsid was first established with *β*-galactosidase, and Peptide-N-Glycosidase F (PNGase F), an enzyme widely used in glycomics and in urgent need of rapid and cost-efficient immobilization methods^32–34^, was immobilized using this system. Characterizations of the immobilized PNGase F indicated high retention of activity, easy recycling, and good stability that sustained five repeated uses. This system uses the naturally available T4 capsid as the nanoparticle-type of supporting material and operates with a simple self-assembly immobilization procedure, which makes it a promising enzyme immobilization technology that could remarkably reduce the cost.

## Results

### *β*-galactosidase immobilized on phage T4 capsid through self-assembly

**Figure 1:**
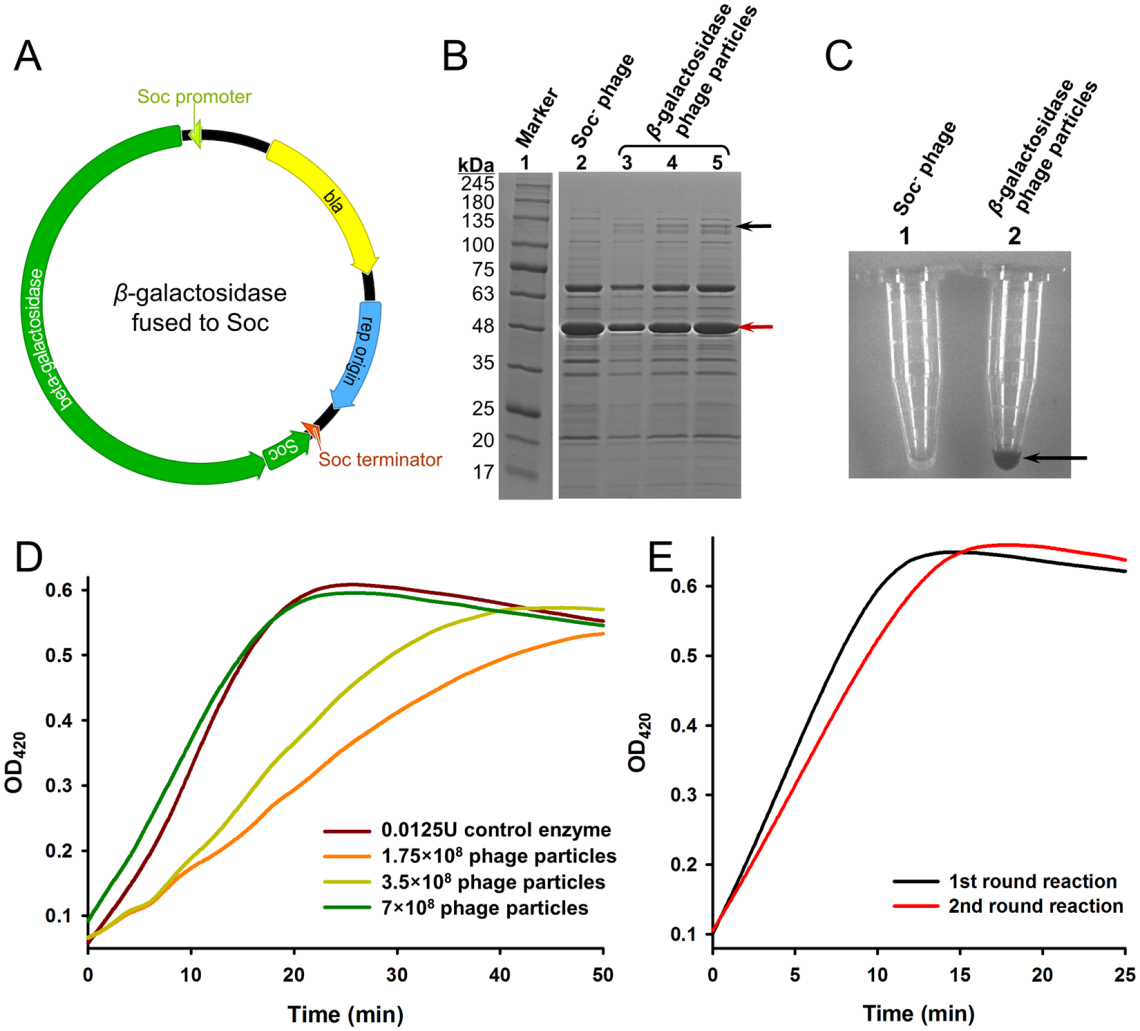
Immobilization of *β*-galactosidase on phage T4 capsid. (**A**) Schematic of the *β*-galactosidase immobilization plasmid. The circular plasmid DNA molecule is represented by a circle. The *β*-galactosidase and Soc genes are represented by green ribbons and the expression directions are indicated as arrow shapes. Soc promoter, Soc terminator, ampicillin antibiotic resistance gene (bla), and the replication origins are indicated. (**B**) SDS-PAGE analysis of the phage particles immobilized with *β*-galactosidase. 5 × 10^8^, 10^9^ and 2 × 10^9^ phage particles assembled with *β*-galactosidase-Soc fusion protein (lanes 3–5) were treated with SDS-PAGE loading buffer (containing 3% SDS), boiled for 10 min, analyzed on a 4–20% gradient SDS-PAGE gel, and stained with Coomassie Brilliant Blue. Lane 1 is the protein molecular weight standard marker, with the molecular weight (in kDa) of each band indicated by a number. Line 2 is 2 × 10^9^ Soc^−^ phage particles treated in the same way as the control. The positions of *β*-galactosidase-Soc fusion protein and T4 gp23 are indicated by black and red arrows, respectively. The image was cropped from different parts of the same gel, and the full-length gel is presented in Supplementary Fig. S1. (**C**) Enzymatic activity of the immobilized *β*-galactosidase. The arrow indicated color change of the reaction mixture in one of the Eppendorf tubes. (**D**) Quantification of immobilized *β*-galactosidase activity. Curves were drawn with the mean of 3 parallel reaction wells. (**E**) Activity of the recycled *β*-galactosidase. After one round of reaction with ONPG for 25 min, phage particles (7 × 10^8^) immobilized with *β*-galactosidase were recycled by centrifugation for the 2^nd^ round of reaction. Curves were drawn with the mean of 3 parallel reaction wells.

The concept of immobilization of enzyme on phage T4 capsid through self-assembly was established using an enzyme whose activity was easy to detect, *β*-galactosidase. The *β*-galactosidase immobilization plasmid was carefully designed and had the following features: (1) the *β*-galactosidase coding sequence was inserted into the upstream sequence of *soc* gene to form an open reading frame (ORF) of a *β*-galactosidase-Soc fusion protein; (2) the *β*-galactosidase-Soc ORF was put under the regulation of Soc promoter and terminator^35,36^; (3) the replication origin was derived from the high copy number vector pUC18, resulting in ~100 copies of the plasmid per *E. coli* cell^37^; (4) the *bla* gene was included in the sequence so that the plasmid was properly maintained in the *E. coli* cells when incubated with antibiotic ampicillin (Fig. 1A). These combined features should lead to stable presence of *β*-galactosidase-Soc ORF in the *E. coli* cells transformed with the plasmid, and when infected with Soc^−^ T4 phage whose *soc* gene was knocked out^29^, the *β*-galactosidase-Soc fusion protein should express as the alternative of Soc protein and assemble on the capsid.

The plasmid was transformed into the *E. coli* strain P301 that well suited for T4 phage infection and amplification^38^. The cells were grown into the log phase and infected with Soc^−^ T4 phage. After the phage amplification cycle, phage particles were isolated from the incubation mixture by differential centrifugations. SDS-PAGE analysis of the phage particles showed an additional protein band corresponding to the molecular weight of the *β*-galactosidase-Soc fusion protein (125 kDa), which was not present in the control Soc^−^ phage (Fig. 1B). Also, the phage particles showed the activity to transfer colorless X-gal into blue product (Fig. 1C). These results indicated presence of *β*-galactosidase on the phage particles, suggesting immobilization of *β*-galactosidase on the phage capsid during the self-assembly process.

Since the band of the *β*-galactosidase-Soc fusion protein was distinct on the SDS-PAGE gel, the copy number of *β*-galactosidase-Soc was determined by the density volumes of the bands that normalized to the gp23 (930 copies per phage particle) bands present in the respective lane, using laser densitometry. The calculated copy numbers from the three lanes were 83.8, 89.7 and 94.6, respectively. Thus, the mean, 89.4, was taken as the copy number.

The enzymatic activity of immobilized *β*-galactosidase was quantified using *o*-nitrophenyl-*β*-galactoside (ONPG) as the substrate (Fig. 1D). 7 × 10^8^ phage particles immobilized with *β*-galactosidase exhibited the same activity as 0.0125 units of the control *β*-galactosidase. Based on the copy number, it is calculated that the specific activity of immobilized *β*-galactosidase was 1.2 × 10^11^ units per mole, which is at the same level as the control (Sigma commercial production, ≥500 units/mg or ≥5.8 × 10^10^ units per mole).

T4 phage particles can be easily separated from soluble substances by differential centrifugation. After one round of reaction, the *β*-galactosidase immobilized on the phage particles was recycled by centrifugation for the 2^nd^ round of reaction. The recycled *β*-galactosidase maintained similar activity as the 1^st^ round (Fig. 1E).

### A self-assembly approach to immobilize PNGase F

As the concept of immobilization of enzyme on phage T4 capsid through self-assembly has been established, PNGase F was selected as the first application trial of this system. PNGase F is one of the most popular enzymes used in glycomics to study N-glycan modifications, but its preparation has suffered from long production cycles and tedious purification procedures^39–41^. Many coupling chemistries were applied to immobilize PNGase F, however, these traditional immobilization methods employed nonspecific chemical reactions between random amines of PNGase F and supporting materials, and usually resulted in poor orientation uniformity and stability^32,33,42–44^. Preparation and immobilization of PNGase F with rapid and cost-efficient methods is of urgent need.

**Figure 2:**
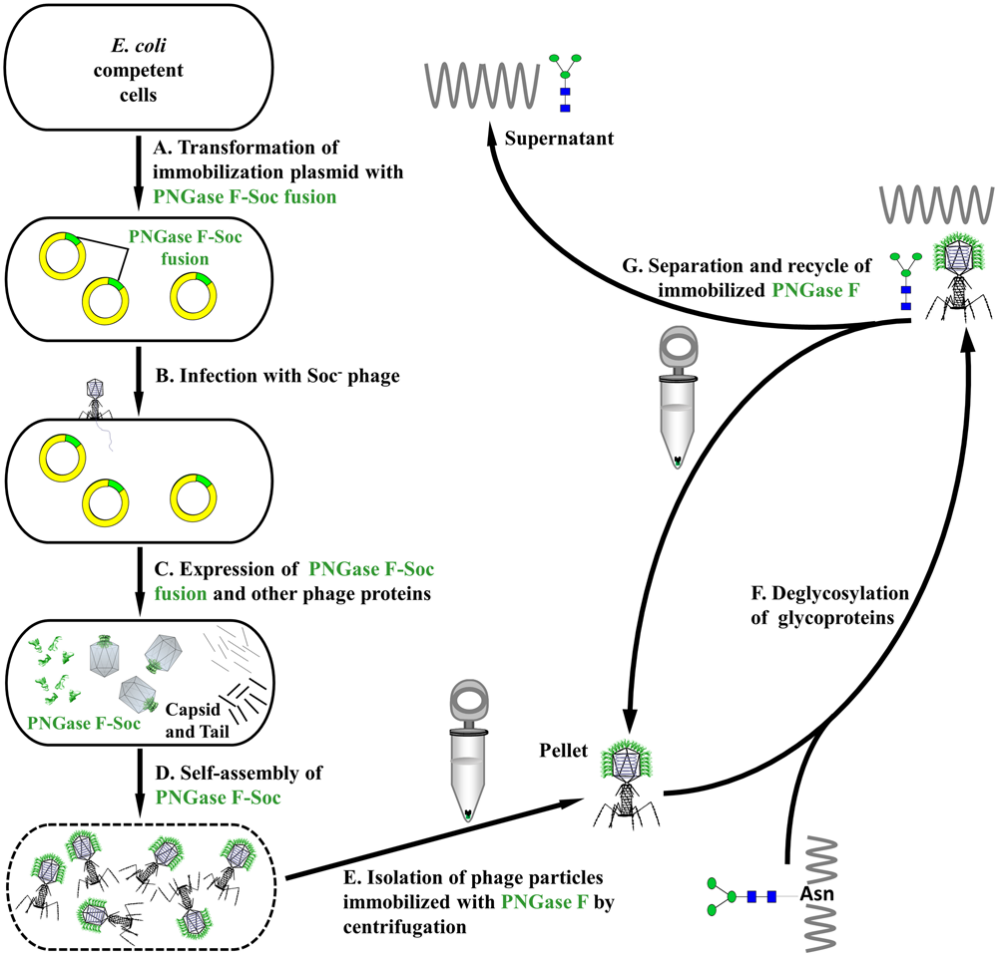
A self-assembly approach to immobilize PNGase F. (**A**) The PNGase F immobilization plasmid was transformed into *E. coli* competent cells. (**B**) *E. coli* cells containing the PNGase F immobilization plasmid were infected by Soc^−^ phage. The Soc^−^ phage had the Soc gene knocked out, so that the phage would not produce endogenous Soc protein. (**C**) The PNGase F-Soc fusion protein was expressed in parallel with other phage proteins that assembled the capsid and tail. The PNGase F-Soc fusion served as the alternative of Soc protein in the phage assembly process. (**D**) At the late stage of phage assembly, the Soc binding sites on the capsid exposed, and the PNGase F-Soc fusion self-assembled on the capsid and immobilized. At the completion of assembly, the *E. coli* cells lysed and phage particles immobilized with PNGase F were released. (**E**) Isolation of the phage particles immobilized with PNGase F at 34,500 *g* for 45 min by centrifugation. (**F**) Deglycosylation of glycoproteins with the PNGase F immobilized on the phage particles. (**G**) Separation and recycle of the immobilized PNGase F (pellet) from the product and/or substrate (supernatant) by centrifugation.

Using the phage T4 immobilization concept established in this study, a self-assembly approach to immobilize PNGase F was designed (Fig. 2). The approach starts with transformation of the immobilization plasmid into *E. coli* cells (Fig. 2A). The plasmid contains the PNGase F-Soc fusion protein gene that is under the regulation of Soc promoter and terminator, and when the *E. coli* cells are infected with Soc^−^ T4 phage (Fig. 2B), the PNGase F-Soc fusion protein expresses in parallel with other phage proteins (Fig. 2C). At the late stage of phage assembly, Soc binding sites on the capsid expose, and the PNGase F-Soc fusion self-assembles on the capsid through its Soc-domain (Fig. 2D). Tail attachment completes the assembly, and the T4 phage particles assembled with PNGase F are released for harvest and isolation (Fig. 2E). Using this approach, PNGase F is immobilized on phage T4 capsid through self-assembly, and the phage particles are conveniently isolated by centrifugation. Moreover, PNGase F immobilized on the phage particles is ready to perform deglycosylation of glycoproteins (Fig. 2F). After the reaction, the enzyme is easily separated from reaction substrates and products by centrifugation, and recycled for repeated uses (Fig. 2G).

### PNGase F was successfully immobilized on phage T4 capsid

**Figure 3:**
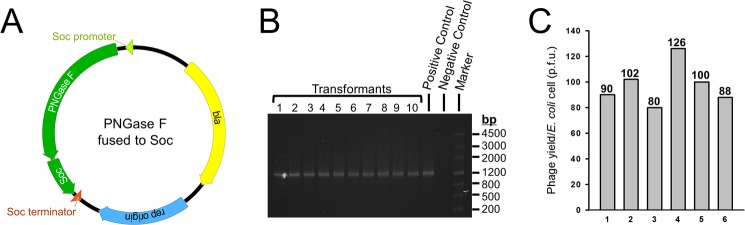
The PNGase F immobilization plasmid construction and maintenance in *E. coli* cells, and phage yield of the immobilization procedure. (**A**) Schematic of the PNGase F immobilization plasmid. The circular plasmid DNA molecule is represented by a circle. The PNGase F and Soc genes are represented by green ribbons and the expression directions are indicated as arrow shapes. Soc promoter, Soc terminator, ampicillin antibiotic resistance gene (bla), and the replication origins are indicated. (**B**) Screening of the transformants for the presence of the PNGase F-Soc ORF. Transformants 1–10 were subjects for colony PCR with primers specific to the PNGase F-Soc ORF, and the PCR products were analyzed by agarose gel electrophoresis. PCR using the PNGase F immobilization plasmid as the template served as the positive control. PCR without a template was used as the negative control. (**C**) Phage yield of the immobilization by self-assembly procedure. The number of phage particles produced from one *E. coli* cell is presented in plaque forming units (p.f.u.). Phage yields from 6 independent experiments (bars 1–6) are shown. The exact number of phage yield of each experiment is indicated on top of each bar.

The plasmid for PNGase F immobilization was designed in the same way as the plasmid for *β*-galactosidase immobilization, except that the PNGase F coding sequence, instead of the *β*-galactosidase coding sequence, was inserted into the upstream sequence of *soc* gene (Fig. 3A). The plasmid was transformed into *E. coli* cells and incubated with antibiotic ampicillin. The property of the plasmid ensured appropriate maintenance of the ORF of PNGase F-Soc fusion in the cells, which was confirmed by an assay screening for the presence of PNGase F-Soc ORF. The result showed that 100% of the transformants were positive (Fig. 3B).

The cells were infected with Soc^−^ T4 phage to start the self-assembly process. After the completion of assembly, phage particles were isolated by differential centrifugations. In order to assess whether assembly of PNGase F-Soc fusion would affect phage amplification, the phage yield from this procedure was calculated by titration. The results showed that, the phage yield was constantly in the range of 100 ± 30 particles per *E. coli* cell, which was a typical yield as compared to natural T4 phage infection events^14^ (Fig. 3C). This suggested that the assembly of PNGase F-Soc fusion did not affect the amplification rate of the phage.

The molecular weight of PNGase F-Soc fusion protein was 45 kDa, which happened to be close to the molecular weight of phage capsid protein gp24^14^, thus SDS-PAGE was not suitable for the identification of PNGase F on the phage particles. The presence of PNGase F on phage particles was verified by mass spectrometry. After trypsin digestion, peptides from the control PNGase F or the phage particles immobilized with PNGase F were analyzed by liquid chromatography-mass spectrum (LC-MS). The results showed a sequence coverage of 75.7% of the control PNGase F digested peptides, and a sequence coverage of 44.9% of the phage particle digested peptides (Supplementary Table S1). Though the sequence coverage of the phage particle digested peptides was relatively low, indication of the presence of PNGase F on the phage particles was still clear. There were two possible reasons why the sequence coverage of the phage particle digested peptides was low. One was that after immobilization, some of the sites on PNGase F for trypsin digestion were no longer accessible, which resulted in incomplete trypsin digestion. The other was that the digested peptides from the immobilized PNGase F were in a pool of a huge number of peptides came from the phage, and the phage peptides were added to the denominator for the sequence coverage calculation.

Although the above possibilities could lead to inaccuracies, an estimation of the copy number of PNGase F on the phage particles from the signal intensities of representative peptides would still be informative. From the data of the phage particle digested peptides, 5 peptides were selected as the representative peptides among the peptides that had the highest contribution and confidence scores (Supplementary Table S2, No. 1–5). These 5 peptides had exact matches in the data of the control PNGase F digested peptides (Supplementary Table S3, No. 1–5). Because the signal intensities of peptide 1, 2, 3 and 5 were too low and buried in the noise, only the signal intensities of peptide 4 were derived (Supplementary Fig. S2). Based on the signal intensity numbers and the amounts of control PNGase F peptides and phage particle peptides loaded onto the column, it was calculated that in average 9 copies of the PNGase F molecule were immobilized on one phage particle (Table 1). Thus, the copy number of PNGase F on the phage particles was estimated as 9.

**Table 1:** Signal intensities of peptide 4 and calculation of PNGase F copy number.

Replicates	Control PNGase F			Phage particle		Calculation	
	m (μg)	No. of molecules	Signal intensity (×10^3^)	No. of phage particles	Signal intensity (×10^3^)	Signal intensity ratio (Control PNGase F vs. Phage particles)	Copy No. (PNGase F on phage particles)
#1	2.5	4.2 × 10^13^	55.2	3 × 10^11^	3.34	16.5	8.4
#2	2.5	4.2 × 10^13^	53.4	3 × 10^11^	3.51	15.2	9.1
#3	2.5	4.2 × 10^13^	52.3	3 × 10^11^	3.91	13.4	10.4

### Enzymatic activity of the immobilized PNGase F

**Figure 4:**
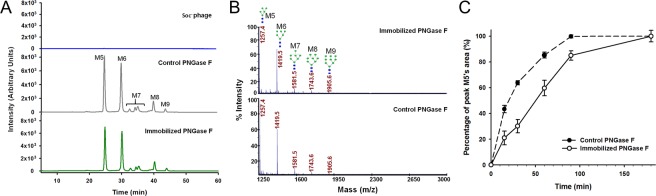
Enzymatic activity of the immobilized PNGase F. (**A**) HPLC profiles of the N-glycans released from RNase B by the immobilized PNGase F (bottom), the control (middle), and the Soc^−^ phage (top). The peaks of the 5 N-glycans (M5 to M9) are indicated. (**B**) Analysis of N-glycans released from RNase B by the immobilized PNGase F (top) and the control (bottom) with MALDI-TOF MS spectra. The structures and mass-to-charge ratios of M5 to M9 are indicated. (**C**) Time curves of the percentages of peak M5’s areas released from RNase B by the immobilized PNGase F and the control. Error bars represent the standard deviation from 3 independent experiments.

Ribonuclease B (RNase B) has five types of oligosaccharides, M5 to M9. If released by N-glycan cutting enzymes, the oligosaccharides should show 7 peaks by HPLC analysis (M7 has 3 isotope glycoforms). Oligosaccharides released from RNase B by PNGase F immobilized on phage particles were analyzed by HPLC and compared with that released by the control PNGase F, and no apparent difference was observed on peak intensities and distributions (Fig. 4A, middle and bottom). Additionally, the control phage particles (Soc^−^ T4 phage without PNGase F-Soc assembly) had no deglycosylation activity (Fig. 4A, top). The released N-glycans were also analyzed by MALDI-TOF MS spectra, and identical peak distributions were obtained from the immobilized PNGase F and the control (Fig. 4B). These results suggested that immobilized PNGase F had the same deglycosylation activity as the control PNGase F.

The peak M5’s areas in the HPLC profiles were used to quantify the enzymatic activity. Same amounts (in mole) of immobilized PNGase F (please note that the amount of immobilized PNGase F was an estimation that based on the estimated copy number) and control PNGase F were used to release the N-glycans from RNase B, and aliquots were removed from the reaction mixture for HPLC analysis at different time points. The results showed that, for both immobilized PNGase F and control PNGase F, the peak M5’s area rapidly increased in the first 60 minutes, and reached the plateau in 90 minutes (Fig. 4C). Although the peak M5’s area of the sample hydrolyzed by the immobilized PNGase F was always lower, the result suggested that the immobilized PNGase F had retained the basic properties of catalysis. Calculated by the percentage of peak M5’s area at the 60 min time point while setting the complete release of M5 as 100%, it was determined that the specific activity of the immobilized PNGase F was 5.3 × 10^11^ units per mole, and the control PNGase F was 7.6 × 10^11^ units per mole (Yugong Biolabs commercial production, ≥20,000 units/mg or ≥7.6 × 10^11^ units per mole). While the calculation of the specific activity of the immobilized PNGase F was based on an estimated copy number, the activity fell in the same range as the control.

### The immobilized PNGase F could be repeatedly used

**Figure 5:**
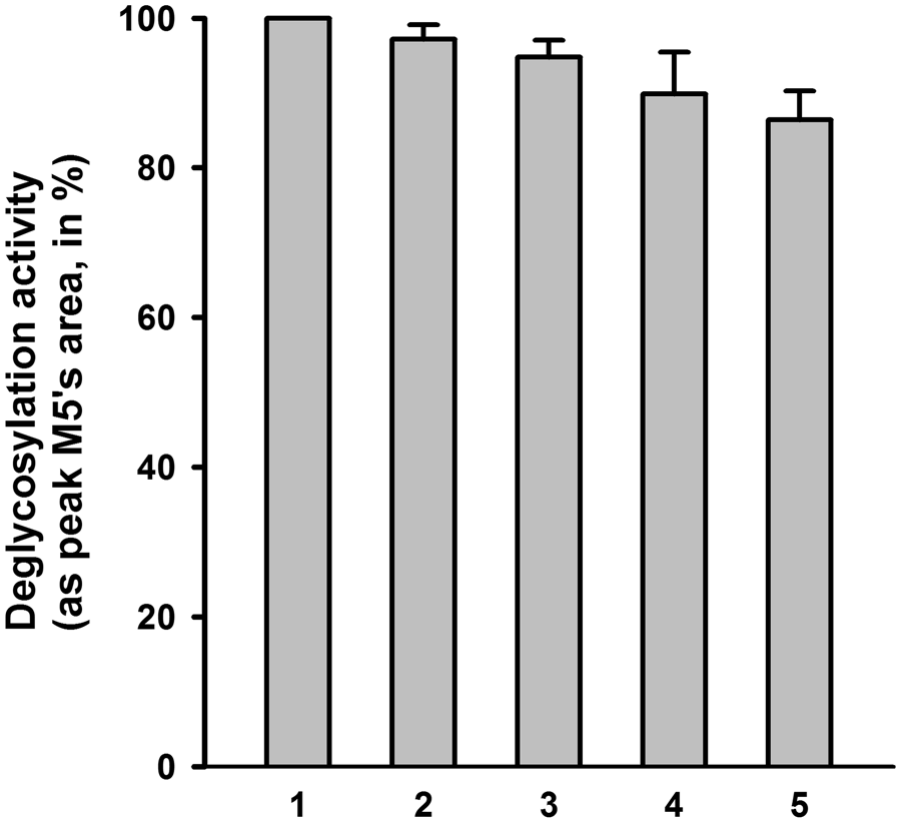
Deglycosylation activities of the immobilized PNGase F in repeated uses. 1.25 × 10^11^ phage particles immobilized with PNGase F (equal to 1 U) were used to digest 10 μg of denatured RNase B at 37 °C for 1 h. After the reaction, the immobilized PNGase F was recycled in the pellet by centrifugation at 34,500 *g* for 45 min at 4 °C. The supernatant was used to analyze the deglycosylation activity by quantification of the released M5. The immobilized PNGase F in the pellet was washed with 1 ml buffer containing 50 mM Tris-HCl (pH 7.5) and 5 mM MgCl_2_ for 3 times, and then used for another round of digestion of 10 μg RNase B. The deglycosylation activities of round 1–5 (indicated under the x-axis) are presented as the peak M5’s areas normalized to the first round of digestion (set at 100%). Error bars represent the standard deviation from 3 independent experiments.

The PNGase F was immobilized on phage particles and should be easily recycled from the enzymatic reactions for repeated uses. To test this possibility, one set of the immobilized PNGase F was used to release N-glycans from RNase B, recycled by centrifugation and reused (Fig. 2G). In the experimental settings, exact units of immobilized PNGase F just enough for the substrate amount were used for the first digestion, and then recycled to digest the same amounts of the substrate in the repeated uses. The recycled PNGase F was buffer-washed for 3 times before each reuse, and the peak M5’s area from the HPLC profile was used to quantify the enzymatic activity of each time. The results showed that, the immobilized PNGase F could release almost the same amounts of N-glycans during the five times of repeated uses, and only very small gradual reductions of activity were seen (Fig. 5). Considering the inevitable loss of phage particles in the process of recycle and wash, this result indicated that the immobilized PNGase F could be repeatedly used for more than 5 times with the same enzymatic activity.

### The immobilized PNGase F was readily applicable for deglycosylation of different substrates

**Figure 6:**
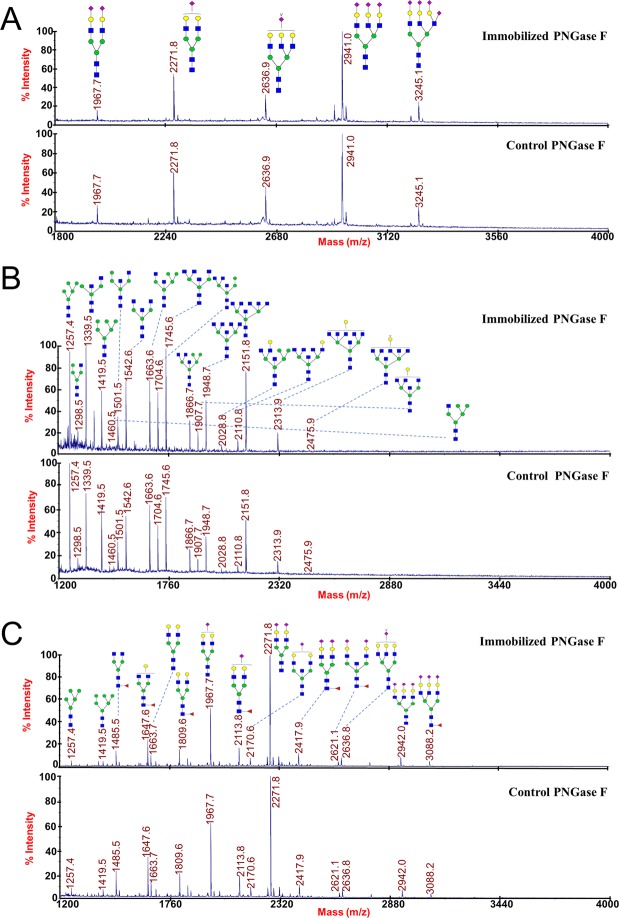
Deglycosylation of different substrates by the immobilized PNGase F. N-glycans released from fetuin (**A**), ovalbumin (**B**) and human serum (**C**) by the immobilized PNGase F (top) and the control (bottom) were analyzed with MALDI-TOF MS spectra. The structures and mass-to-charge ratios of the N-glycans are indicated.

After being validated by RNase B which is a high-mannose glycoprotein, the applicability of the immobilized PNGase F was further evaluated by cleavage of glycoproteins with highly-sialylated, hybrid or complex N-glycans. Fetuin possesses 5 highly sialylated N-glycans; ovalbumin is a well-studied glycoprotein with hybrid oligosaccharides; N-glycans from human serum are a complex N-glycan sample^45–47^. When these proteins were used as the substrates, the MALDI-TOF MS spectra results demonstrated identical peak distributions from the immobilized PNGase F and the control (Fig. 6), indicating that the immobilized PNGase F had the same deglycosylation capacity as the control PNGase F to release different types of N-glycans.

## Discussion

Enzyme-based biocatalysts are more and more widely accepted in this era when environmental concerns are increasingly raised^5^. Immobilized forms of enzymes are often preferred due to their prolonged stability and ease of recycling that curtails the redundant downstream processing. However, the supporting materials for immobilization and the technology required to apply fixation of the enzymes greatly increased the cost of immobilized enzymes, and limited their uses^7^. The novel immobilization system developed in this study used the naturally available T4 capsid as the supporting material, and achieved enzyme fixation with a simple self-assembly procedure. These combined features could remarkably reduce the cost, making this system promising for many enzyme immobilization applications.

**Figure 7:**
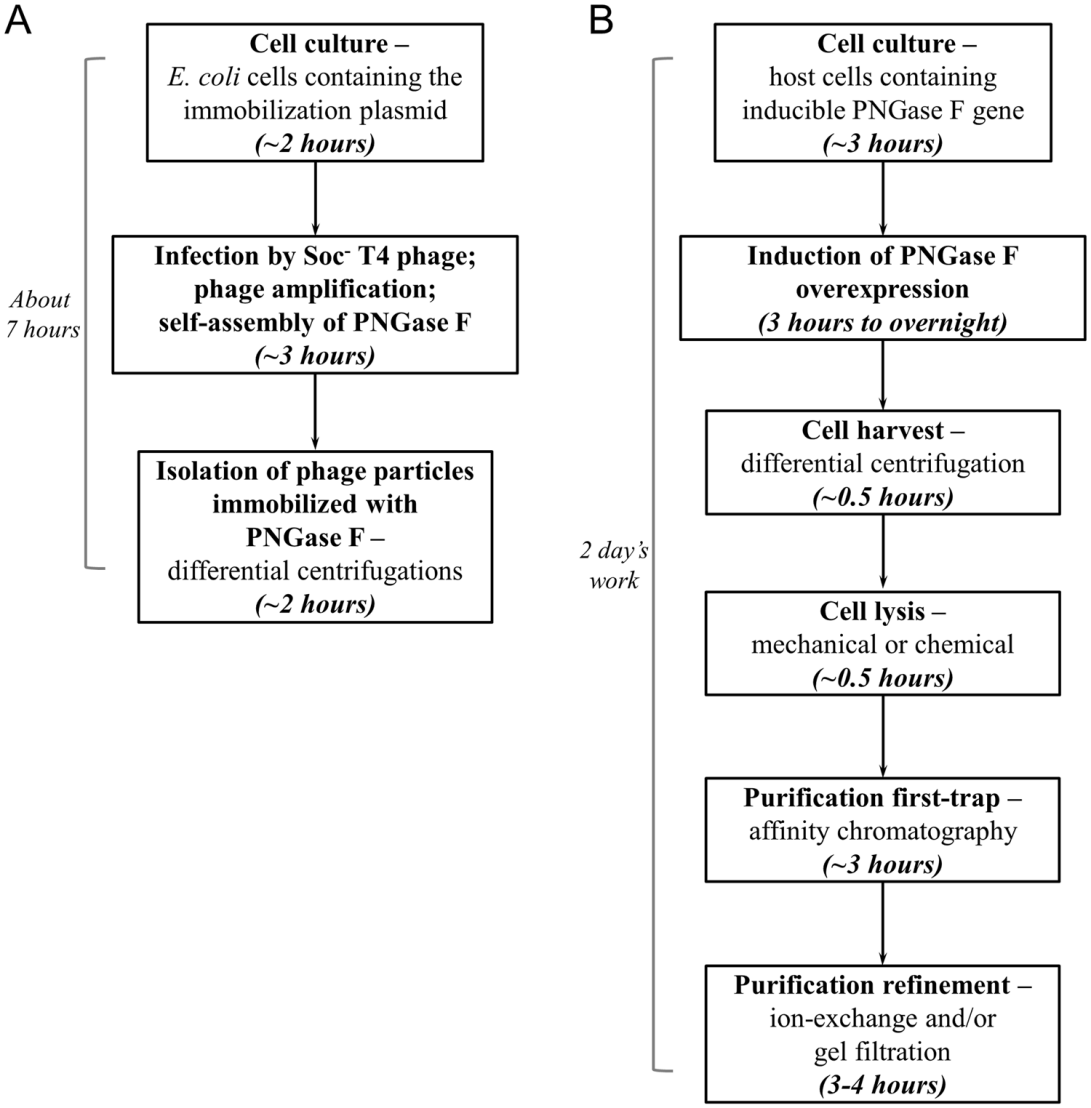
Comparison of the immobilization method and traditional method to obtain PNGase F. (**A**) Flow chart showing the procedure of the immobilization method. The cell culture of *E. coli* containing the immobilization plasmid was grown for 2 h, and infected by Soc^−^ phage at a multiplicity of infection of 1. After infection, the phage amplified and PNGase F self-assembled. 3 h of incubation time ensured complete amplification and assembly of phage in the culture. The phage was isolated from the culture by differential centrifugations. The total time required was 7 h. (**B**) Flow chart of the procedure to produce PNGase F in the traditional way. Cell culture with the inducible PNGase F gene was grown to an appropriate density and PNGase F overexpression was induced for a time period ranging from 3 h to overnight. The cells were harvested and lysed, followed by purification steps including affinity chromatography, ion-exchange and/or gel filtration. Two days were required to produce PNGase F in the traditional way.

Immobilization of PNGase F was a good example. By using this self-assembly system, production and simultaneous immobilization of PNGase F was finished in 7 hours with a procedure consisted of cell culturing, phage infection and isolation of the immobilized enzyme (Fig. 7A). The immobilization retained the enzymatic activity well, as indicated by the specific activity similar to the control free PNGase F, and the same capacity to release different types of N-glycans (Figs 4 and 6). The immobilized PNGase F was stable and remained fully active for five repeated uses (Fig. 5).

As a comparison, the traditional way to obtain PNGase F (not even immobilized) was much more complicated (Fig. 7B). PNGase F was usually recombinantly expressed by induction in a suitable host. The induction time ranged from 3 h to overnight. The cells were harvested by differential centrifugation, and lysed by mechanical or chemical means. PNGase F in the cell lysate was trapped on an affinity media through the purification tag fused to it, and eluted for refining purification steps that usually include ion-exchange and gel filtration. The total time to produce a batch of PNGase F was no less than 2 days. Researchers have used covalent binding to chemically immobilize the purified PNGase F onto supporting materials^43,48^. Not only the procedures were tedious, but also the chemical binding included random modifications of free amino or carboxyl groups on PNGase F that would block the activity. Affinity immobilization by fusing the glutathione-S-transferase to PNGase F were also used, however the immobilization procedure was laborious that took up to 14 hours^32,33^. Immobilization of PNGase F through the self-assembly system was apparently simpler and more cost-efficient.

From the copy number calculations, 89.4 copies of *β*-galactosidase, or 9 copies of PNGase F, was assembled onto one phage particle. Based on these copy number calculations, the specific activities of both the immobilized enzymes were at the same level as the controls (see Results), indicating the enzymatic activities were well retained after the immobilization procedure. Both immobilized enzymes achieved enzymatic performances that were as good as the free controls, and showed the recycle and reuse capacity, suggesting that the procedure as it is now, can serve as a good enzyme immobilization method for *β*-galactosidase or PNGase F.

Considering there were 870 Soc binding sites per phage particle^18^, the procedure could be further improved by increasing the assembly rate (number of copies per particle). For both enzymes, the assembly rates were relatively low comparing to the number of available binding sites. The possible cause for the low assembly rate could be that there was not enough Soc fusion protein present in the cytosol at the time of assembly. Increasing the expression level of the Soc fusion protein, e.g. re-designing the promoter region of the Soc fusion expression construct, would improve the immobilization. It has to be pointed out that, the copy number of *β*-galactosidase was 10 times higher than that of the PNGase F, but both immobilized enzymes performed well on activity and recycling. This suggested that for *β*-galactosidase and PNGase F, the assembly rate was not a key factor for the immobilization performance. If the immobilization system is applied to an enzyme for which the assembly rate is indeed a key to the enzymatic performance, systematic studies specifically towards the particular enzyme will have to be carried out to optimize the procedure and increase the assembly rate.

The major difference and advantage of the self-assembly immobilization system is that it finishes enzyme production and immobilization in one operation. There is no need to separately produce the enzyme and prepare the supporting material, and then perform the fixation reaction. The supporting material is natural and self-amplified, and the enzyme is self-assembled, making the system simple with low cost. The enzyme is immobilized onto a nanoparticle-type of carrier through affinity binding, which facilitates good retention of the activity^7,10,13^. Based on the intrinsic principle of the system, which is the phage T4 assembly process^14^, enzymes that could be directly applied to this system should have the following properties: (a) should be able to actively express in *E. coli*; and (b) should function without the requirement of other subunits. This is in agree with the successful immobilization of *β*-galactosidase and PNGase F. Both *β*-galactosidase and PNGase F can be expressed actively in *E. coli*, and their functions are independent of any other subunit^40,49,50^. Being the most preferred recombinant expression system, *E. coli* has been used for the production of a large number of important industrial enzymes^3,51–53^. Among them, those independent of other subunits are readily applicable to this immobilization system. For enzymes that need other subunits, additional studies have to be done to properly assemble additional subunits after one subunit of the enzyme has been attached onto the phage capsid.

Using this self-assembly system, preparation of immobilized enzyme becomes simple and fast. As indicated by our results, recycling of the immobilized PNGase F by centrifugation led to only ~3% loss of activity each time (Fig. 5). The loss percentage could be further lowered by optimizing the recycling method, such as using filtration instead of centrifugation. It is also possible to plant the phage particles onto solid support surfaces through the specific binding of the T4 tail fibers to lipopolysaccharide to construct column- or chip- type of immobilized enzymes^14^. Exploration into these directions would optimize the self-assembly enzyme immobilization system for various industrial needs. In addition, the Hoc binding sites on the T4 capsids could also be utilized, inferring the possibility of immobilizing two enzymes as Soc- and Hoc- fusions on the same capsid. These potentials would add more value of this system in the future applications.

## Methods

### Construction of *β*-galactosidase immobilization plasmid

The skeleton of the immobilization plasmid, including the replication origin and an antibiotic resistance gene, was derived from vector pUC18. Using pUC18 plasmid as the template, a DNA fragment with the bla (ampicillin resistance) gene and the replication origin sequence was PCR-amplified (Monad Biotech Co. Ltd., Wuhan, China). The forward PCR primer (sequence: CCCAGATCTCATATGGTGCACTCTCAG) included an 18 bp nucleotide sequence upstream of the bla gene; a BglII restriction site was added to the 5′-end. The reverse PCR primer (sequence: CTAAGATCTGGCTGCGGCGAGCGGTAT) included an 18 bp nucleotide sequence downstream of the replication origin and a BglII restriction site at the 5′-end as well. The amplified DNA fragment (2165 bp) contained the bla gene and the replication origin, and served as the skeleton of the immobilization plasmid.

The cloning/expression region of the immobilization plasmid was synthesized as one DNA fragment (Genewiz Inc., Jiangsu, China). We put *soc* gene (GenBank: NP_049644.1) under the control of *soc* promoter and *soc* terminator. A multiple cloning sites region (MCS) was placed upstream of the *soc* gene to facilitate the insertion of exogenous genes. A BglII restriction site was placed at both ends of the synthesized DNA fragment.

The PCR amplified skeleton fragment and the synthesized DNA fragment of the cloning/expression region were digested by BglII restriction enzyme (Monad Biotech Co. Ltd.) and ligated into a circular DNA molecule. The ligated DNA was transformed into *E. coli* DH5α competent cells for plasmid amplification. The plasmid DNA was purified using the Axygen AxyPrep Plasmid Miniprep Kit (Axygen Biosciences, Inc., Union City, CA, USA). Accuracy of the plasmid DNA was ascertained by DNA sequencing (Genewiz Inc.).

The gene of *β*-galactosidase (GenBank: WP_000177906.1) was synthesized with restriction sites NheI and HindIII placed at the immediate upstream and downstream flanking regions, respectively (Genewiz Inc.). Synthesized DNA was digested with NheI and HindIII restriction enzymes (Monad Biotech Co. Ltd.) and ligated into the MCS of the immobilization plasmid, resulting in the fusion of *β*-galactosidase to the N-terminus of Soc. The ligated DNA was transformed into *E. coli* DH5α competent cells for plasmid amplification. The amplified immobilization plasmid with the *β*-galactosidase-Soc fusion was purified (Axygen Biosciences, Inc.) and sequenced for accuracy (Genewiz Inc.).

### Immobilization of *β*-galactosidase through self-assembly

The immobilization plasmid with the *β*-galactosidase-Soc fusion was transformed into *E. coli* P301 competent cells (the *E. coli* P301 strain was a kind gift from Dr. Venigalla B. Rao’s lab in The Catholic University of America, Washington D.C., USA). The cells were grown to 3–4 × 10^8^/ml, infected with the Soc^−^ phage (multiplicity of infection of 1) and incubated at 37 °C shaking for 3 h (the Soc^−^ phage was a kind gift from Dr. Venigalla B. Rao’s lab). At the end of incubation all the cells were infected to amplify the phage. The *β*-galactosidase-Soc fusion protein was self-assembled onto the phage capsids through the Soc binding sites.

### Isolation of T4 phage particles immobilized with enzyme

Phage particles were harvested from the incubation culture by centrifugation at 34,500 *g* for 45 min at 4 °C. The pellet was resuspended in 1/10 volume of Pi-Mg buffer [26 mM Na_2_HPO_4_, 68 mM NaCl, 22 mM KH_2_PO_4_, and 1 mM MgSO_4_ (pH 7.5)]. 10 μg/ml DNase I (Amresco LLC, Solon, OH, USA) was added to the phage suspension and chloroform (5–6 drops per ml) was added to completely lyse the bacteria. The suspension was kept shaking at 37 °C for 30 min and centrifuged at 4,000 *g* for 10 min to remove residual cell debris. The supernatant containing the phage particles was pelleted by centrifugation at 34,500 *g* for 45 min and resuspended in a buffer containing 50 mM Tris-HCl (pH 7.5) and 5 mM MgCl_2_. The phage particles were titered on *E. coli* P301, quantified, and stored at 4 °C.

### Enzymatic activity test of immobilized *β*-galactosidase

*β*-galactosidase produces 5-bromo-4-chloro-3-indole (blue) from colorless 5-bromo-4-chloro-3-indole-beta-d-galactoglycosidese (X-gal). 10^9^ phage particles immobilized with *β*-galactosidase or 10^9^ Hoc^−^Soc^−^ phage particles were added to a reaction tube and mixed with 1 μg X-gal (iScience, Yugong Biolabs, Inc.). After incubation for 30 min at 37 °C, color change of the reaction mixture was observed and recorded. Activity of *β*-galactosidase was quantified using *o*-nitrophenyl-*β*-galactoside (ONPG) as the substrate. The 200 μl reaction mixture containing 625 μM ONPG, 100 mM phosphate buffer, pH 6.8, 1 mM MgCl_2_, and various amounts of phage particles immobilized with *β*-galactosidase was incubated at 37 °C in a microplate spectrophotometer (Multiskan GO, Thermo Scientific Inc.). Absorbance of OD_420_ was read with 2-min intervals. Every reaction was performed in 3 parallel wells. Commercial *β*-galactosidase (Sigma-Aldrich, St. Louis, MO, USA) was used as the control.

### Calculation of *β*-galactosidase copy number on phage particles

After Coomassie blue staining and destaining, the SDS-PAGE gel was scanned by a laser densitometer (PDSI, GE Healthcare Corp.) and the density volumes of the displayed *β*-galactosidase-Soc fusion protein bands and the internal control band, T4 gp23, were quantified as described^24^. Each lane was individually quantified so that the copy number of the assembled enzyme was obtained in comparison with the gp23 internal control for which the copy number was established to be 930 per particle.

### Construction of PNGase F immobilization plasmid

The gene of PNGase F (GenBank: AF165910.1) was synthesized with restriction sites EcoRI and NotI placed at the immediate upstream and downstream flanking regions, respectively (Genewiz Inc.). Synthesized DNA was digested with EcoRI and NotI restriction enzymes (Monad Biotech Co. Ltd.) and ligated into the MCS of the immobilization plasmid, resulting in the fusion of PNGase F to the N-terminus of Soc. The ligated DNA was transformed into *E. coli* DH5α competent cells for plasmid amplification. The amplified immobilization plasmid with the PNGase F-Soc fusion was purified (Axygen Biosciences, Inc.) and sequenced for accuracy (Genewiz Inc.).

### Immobilization of PNGase F

The immobilization plasmid with the PNGase F-Soc fusion was transformed into *E. coli* P301 competent cells. The transformants were screened for the presence of PNGase F-Soc ORF by colony PCR using primers specific to the PNGase F-Soc ORF sequence. The cells of positive transformants were grown to 3–4 × 10^8^/ml, infected with the Soc^−^ phage (multiplicity of infection of 1) and incubated at 37 °C shaking for 3 h. The PNGase F-Soc fusion protein was self-assembled onto the phage capsids through the Soc binding sites. The phage particles were isolated as described above, titered on *E. coli* P301, quantified, and stored at 4 °C.

### Confirmation of the immobilization of PNGase F

#### Trypsin digestion

1.2 × 10^12^ phage particles immobilized with PNGase F or 10 μg control PNGase F (NOVA, Yugong Biolabs, Inc.) in 1 ml H_2_O was denatured by boiling at 100 °C for 10 min and cooling at 4 °C for 5 min. Urea was added to a final concentration of 8 M and the sample was incubated at 37 °C shaking for 1 h. The sample was centrifuged at 12,000 g for 10 min and the supernatant was collected and ultra-filtrated with buffer change in Amicon Ultra-15 centrifugal filters (3 kDa molecular weight cut-off; Millipore, Merck KGaA) to reach a final volume of 500 μl in 50 mM NH_4_HCO_3_ (pH 8.0). 75 μl of 200 mM dithiothreitol (DTT) was added and the sample was shaking at 300 rpm for 1 h at 37 °C, and then 75 μl of 200 mM iodoacetamide (IAA) was added. The sample was then kept at room temperature in the dark for 1 h. Trypsin (Promega Corporation, Madison, WI, USA) was added at a substrate-to-enzyme ratio of 50:1 (w/w) for the control PNGase F, or 1.0 × 10^−11^ μg per particle for the phage particles, mixed by brief vortex, and incubated at 37 °C for 24 h. The digestion mixture was diluted with 0.5 ml of 0.1% Trifluoroacetic acid (TFA; Merck KGaA, Darmstadt, Germany) and extracted by a C18 solid phase extraction column (Waters Corporation, Milford, MA, USA) equilibrated with 0.1% TFA. The column was washed with 4.0 ml of 0.1% TFA and the peptides were eluted by 1.0 ml of the elution buffer [50% acetonitrile (ACN), 0.1% TFA]. Peptides in the eluate were dried under vacuum for LC-MS analysis.

#### LC-MS

The LC-MS system consisted of a NanoLC Ultra System (Eksigent Technologies, Dublin, CA, USA), a TripleTOF 5600 System (AB SCIEX, Framingham, MA, USA) equipped with a trap column (150 μm I.D. × 10 mm L.; C18, 3 μm, 100 Å; Proteomics Front, Beijing, China) and a separation column (75 μm I.D. × 150 mm L., C18, 3 μm, 100 Å; Proteomics Front). The dried peptides were dissolved in 20 μl of solvent A [5% ACN, 0.1% Formic Acid (FA)], and a 5-μl aliquot was injected into the trap column at a flow rate of 2.0 μl/min. The analytical separation was conducted at a flow rate of 300 nl/min. After a 4-min wash with 5% solvent B (95% ACN, 0.1% FA), a 45-min linear gradient was run with 5–80% solvent B followed by a 5-min linear gradient with 80–95% solvent B. The eluate was directly evaporated at 150 °C with a nitrogen stream of 3 l/min, and the ion spray voltage was set at 2.3 kV. MS was operated in the positive-ion mode with a mass range of 400–4000 m/z. MS/MS was acquired in an automated data-dependent acquisition mode with charge numbers of 2–4 and a mass range of 100–2000 m/z. The data were analyzed with PeakView 1.2 software (AB SCIEX). The sequence coverages were analyzed with ProteinPilot^TM^ software using Database uniprot_sprot_20121010 specifically towards Accession # sp|P21163|PNGF_ELIMR^54^.

#### Calculation of PNGase F copy number on phage particles

The signals of the representative peptides were identified to retrieve the signal intensities. The amounts of control PNGase F peptides and phage particle peptides loaded onto the LC column were calculated from the above experimental procedures to be 2.5 μg and 3 × 10^11^ particles, respectively. The molar ratio of PNGase F (control vs. immobilized) equals the ratio of signal intensities (control PNGase F peptide vs. phage particle peptide).

### PNGase F enzymatic activity detection and analysis

#### N-glycan hydrolysis and extraction

10 μg of glycoprotein (RNase B, bovine fetuin, and chicken ovalbumin were obtained from Sigma-Aldrich) or 1 μl of human serum sample (obtained from the Tongji Medical College of Huazhong University of Science and Technology) was denatured with 1X Denaturing Buffer (0.5% SDS, 40 mM DTT) at 100 °C for 10 min. Then 10 μl 10% NP-40, 10 μl 10X Reaction Buffer (500 mM sodium phosphate, pH 7.5), 2.5 × 10^11^ phage particles immobilized with PNGase F or 0.1 μg control PNGase F, and water were added to constitute a 100-μl reaction mixture. The reaction mixture was incubated at 37 °C for 1 h with gentle vibration to release the N-glycans. The N-glycans were extracted with porous graphitized carbon (PGC) solid phase extraction. The PGC cartridge (Sigma-Aldrich) was equilibrated with 3.0 ml of the equilibrium buffer (5% ACN, 0.1% TFA). The reaction mixture was loaded on the cartridge and washed with 3.0 ml of the equilibrium buffer to remove the salts and proteins. The N-glycans were eluted with 1.0 ml of the elution buffer (40% ACN, 0.1% TFA), and dried under vacuum for HPLC and MALDI-TOF analyses.

#### HPLC analysis

The dried N-glycans were dissolved in 14 μl of Dimethyl sulfoxide (DMSO) and 6 μl of acetic acid, and 1 mg 2-aminobenzoic acid (2-AA) and 2.7 mg 2-picoline borane (2-PB) were quickly added for 2-AA derivatization^55^. The mixture was incubated at 65 °C for 2 h, diluted with 0.5 ml of 1-butanol/ethanol/H_2_O (4:1:1, v/v/v) and purified with a microcrystalline cellulose (MCC) column (Sigma-Aldrich)^56^. The MCC column was equilibrated with 3 ml of 1-butanol/ethanol/H_2_O (4:1:1, v/v/v), and the diluted N-glycans were loaded and washed with 3 ml of the equilibrium buffer, followed by elution with 1 ml of ethanol/H_2_O (1:1, v/v). The eluate was dried under vacuum and analyzed with an HPLC analytical system (Shimadzu, Nakagyo-ku, Kyoto, Japan) consisted of two LC-20AD pumps, a SIL-20AC auto sampler, and a RF10AXL fluorescence detector. The 2-AA-derivatized N-glycans were resolved in Solvent A (50 mM ammonium formate) and analyzed on an Amide 80 column (Tosoh, Tokyo, Japan; 4.6 mm I.D., 250 mm) set at 40 °C. After sample injection, the column was eluted with the mixture of Solvent A and B containing 68% Solvent B (100% ACN) for 5 min at a flow rate of 1.0 ml/min, and then a 60-min linear decreasing gradient of 68–43% Solvent B. The excitation/emission wavelengths of the fluorometric detection were λ_ex_ = 360 nm and λ_em_ = 419 nm for 2-AA derivatives.

#### MALDI-TOF analysis

The extracted and dried N-glycans containing sialic acid were dissolved in 25 μl of a DMSO solution containing 1 M methylamine hydrochloride and 0.5 M N-methylmorpholine, and then mixed with 25 μl of PyAOP solution (50 mM trispyrolidinophosphonium hexafluorophosphate in DMSO). The reaction mixture was placed in dark at room temperature for 40 min, diluted with 0.5 ml of 1-butanol/ethanol/H_2_O (4:1:1, v/v/v), and purified by the MCC column as described above. The eluate from the MCC column was dried under vacuum, and dissolved in 10 μl of 50% ACN. 1 μl of the dissolved sample was mixed with 1 μl of freshly-made DHB solution (10 mg/ml 2,5-dihydroxybenzosa acid in 50% ACN containing 5 mM NaAc) and loaded onto the MALDI plate. N-glycans from RNase B or ovalbumin that did not contain sialic acid were directly dissolved in 10 μl of 50% ACN for MALDI-TOF analysis. The MALDI-TOF MS spectra were obtained with 5800 MALDI-TOF/TOF (Applied Biosystems/MDS Sciex, Concord, Canada) equipped with an Nd:YAG laser with 355 nm wavelength of <500 ps pulse and 200 Hz repetition rate. The spectra were obtained in the reflectron mode and were accumulated with 1200 laser shots. The MS data were further analyzed using Data explorer 4.0 (Applied Biosystems/MDS Sciex) and the structural models of N-glycans were constructed with GlycoWorkbench 2.1^47,57^.

#### Enzymatic activity determination

1.1 × 10^−12^ moles of immobilized PNGase F (equal to 7 × 10^10^ phage particles) or control PNGase F (equal to 0.04 μg) were used to digest 10 μg of denatured RNase B. Aliquots were removed from the reaction mixture at time points 15 min, 30 min, 60 min, 90 min and 180 min. The N-glycans released were extracted and analyzed by HPLC as described above. The peak M5’s areas in the HPLC profiles were normalized to the complete release of M5 (time point 180 min). The percentage of peak M5’s area at the time point 60 min was used for the unit determination. In this study, the PNGase F’s activity was defined as follows: one unit of PNGase F will catalyze the deglycosylation of 10 μg of denatured RNase B in 60 min at pH 7.5 at 37 °C.

## Supplementary information

SUPPLEMENTARY INFO

## Data Availability

All data generated or analyzed during this study are included in this published article (and its Supplementary Information files).
