# Supplementary material for: Bacteriophage T4 capsid as a nanocarrier for Peptide-N-Glycosidase F immobilization through self-assembly
Source: Sci Rep. 2019 Mar 19;9:4865. doi: 10.1038/s41598-019-41378-9 (PMC6424964; doi:10.1038/s41598-019-41378-9)
Supplement: Supplementary file 1 — SUPPLEMENTARY INFO [file 41598_2019_41378_MOESM1_ESM.pdf]

Supplementary Information

Bacteriophage T4 capsid as a nanocarrier for Peptide-N-Glycosidase F immobilization through self-assembly

Liang Zhang[1,2,†], Pei Wang[1,3,4,5,†], Chang Wang[2], Yike Wu[2], Xiaojun Feng[2], He Huang[4,6], Lujing Ren[4,7], Bi-Feng Liu[2], Song Gao[1,3,4,*], and Xin Liu[2,*]

[1]Jiangsu Key Laboratory of Marine Bioresources and Environment, Huaihai Institute of Technology, Lianyungang 222005, China

[2]Britton Chance Center for Biomedical Photonics at Wuhan National Laboratory for Optoelectronics – Hubei Bioinformatics & Molecular Imaging Key Laboratory, Systems Biology Theme, Department of Biomedical Engineering, College of Life Science and Technology, Huazhong University of Science and Technology, Wuhan 430074, China

[3]Jiangsu Marine Resources Development Research Institute, Lianyungang 222005, China

[4]Co-Innovation Center of Jiangsu Marine Bio-industry Technology, Huaihai Institute of Technology, Lianyungang 222005, China

[5]Yugong Biolabs, Inc., Lianyungang 222000, China

[6]School of Pharmacy, Nanjing Tech University, Nanjing 211816, China

[7]College of Biotechnology and Pharmaceutical Engineering, Nanjing Tech University, Nanjing 211816, China

[*]Correspondence and requests for materials should be addressed to S.G. (email: gaosong@hhit.edu.cn) or X.L. (email: xliu@mail.hust.edu.cn)

[†]These authors contributed equally to this work.

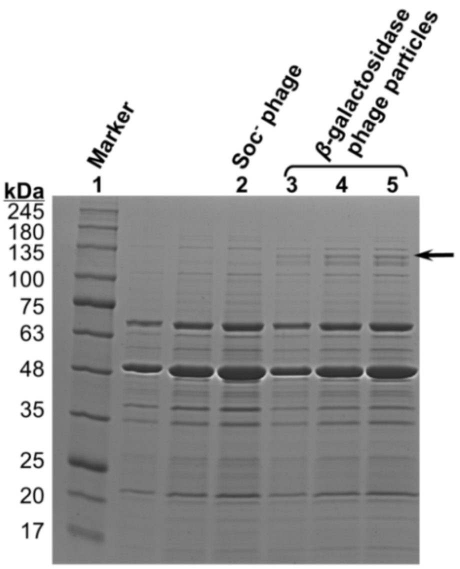

Figure S1.

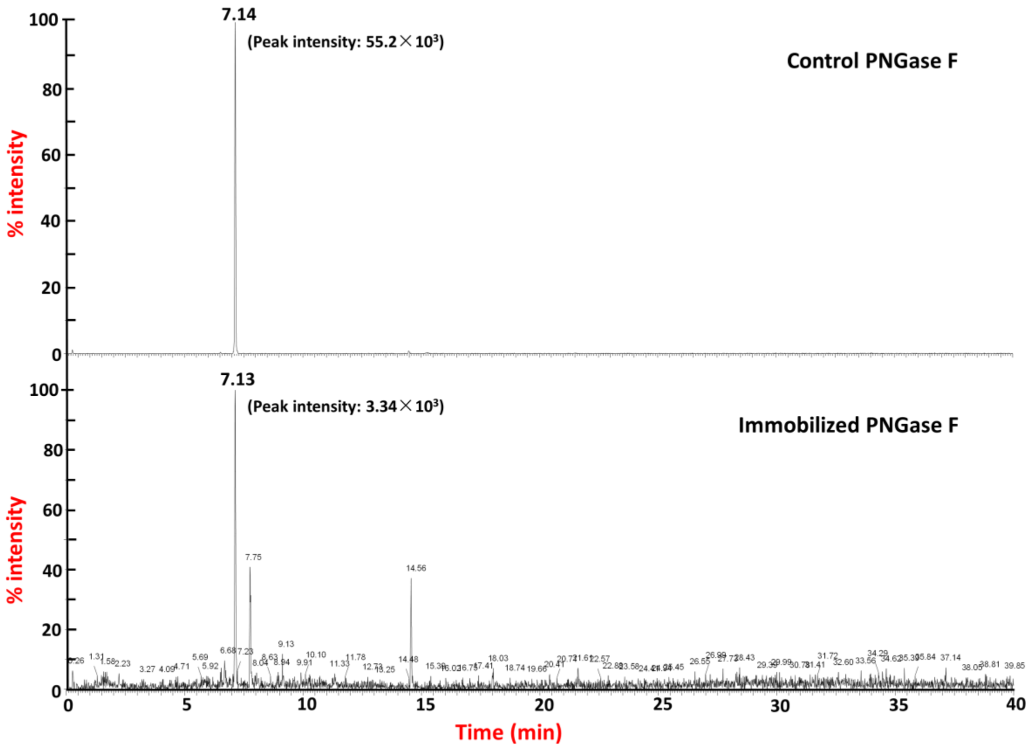

Figure S2. LC-MS data extracted for 511.73 (m/z) peptides.

Signals of peptides with 511.73±0.5 (m/z) (representing the peptide 4, refer to Tables S2 and S3 for m/z calculation) were extracted from the total ion chromatograms. Spectra of the extracted signals from the trypsin digested control PNGase F (top) and phage particles immobilized with PNGase F (bottom) are presented. Signals at the retention time of ~7.1 min were identified as from the peptide 4. This is one representative figure of three replicates.

Table S1. Protein detected and sequence coverages

Sample	Sequence coverage	Accession No.	Name	Species	Peptides (95%)
**Control PNGase F**	75.7%	sp|P21163|PNGF_ELIMR	Peptide-N(4)-(N-acetyl-beta-D-glucosaminyl) asparagine amidase F	ELIMR	142
**Phage particles**	44.9%	sp|P21163|PNGF_ELIMR	Peptide-N(4)-(N-acetyl-beta-D-glucosaminyl) asparagine amidase F	ELIMR	10

Table S2. Peptides with highest contribution and confidence scores (Phage particle sample)

No.	Contribution	Confidence	Sequence	Precise molecular weight	z
1	2	99	AGWCPGMAVPTR	1301.623	2
2	2	99	NIQLPTNTEK	1156.607	2
3	2	99	SLLSGNTELK	1060.575	2
4	2	99	SSIDGVPYGK	1021.517	2
5	2	99	YANVYVK	855.4571	2
6	2	99	TCDEWDRYANVYVKNK	2060.856	2
7	2	99	TEKAYLRTTISGWGHAK	1917.929	4

Table S3. Peptides with highest contribution and confidence scores (Control PNGase F sample)

No.	Contribution	Confidence	Sequence	Precise molecular weight	z
1	2	99	AGWCPGMAVPTR	1301.598	2
2	2	99	NIQLPTNTEK	1156.589	2
3	2	99	SLLSGNTELK	1060.564	2
4	2	99	SSIDGVPYGK	1021.507	2
5	2	99	YANVYVK	855.4483	2
6	2	99	ADNTVNIK	873.4775	2
7	2	99	AISSFVIAK	934.55	2
8	2	99	FDIVYGTPDYK	1316.64	2
9	2	99	FITPYWVGTEK	1339.673	2
10	2	99	GCAEWCFR	1084.41	2
11	2	99	GLEIDVTDFK	1135.575	2
12	2	99	IQLPTNTEK	1042.566	2
13	2	99	IVYGTPDYK	1054.536	2
14	2	99	IYTETWLAK	1123.589	2
15	2	99	KNIQLPTNTEK	1284.706	3
16	2	99	NAFGDGLSQSAEGT	1352.601	2
17	2	99	NAFGDGLSQSAEGTF	1499.659	2
18	2	99	NKTTGEWYEIGR	1452.7	3
19	2	99	SIDGVPYGK	934.4803	2
20	2	99	SNTPISAPVVT	1084.576	2
21	2	99	SNTPISAPVVTN	1198.627	2
22	2	99	SSIDGVPY	836.3905	2
23	2	99	SVDFDIVYGTPDYK	1617.77	2
24	2	99	TCDEWDR	980.3639	2
25	2	99	TFQHQLGALGC	1230.586	2
26	2	99	TTGEWYEIGR	1210.549	2
27	2	99	TTISGWGH	857.4033	2
28	2	99	TTISGWGHAK	1056.535	2
29	2	99	TTISGWGHAKPYDAGSR	1802.875	4
30	2	99	YAISSFVIAK	1097.609	2
